# A meta-review of evidence on heart failure disease management programs: the challenges of describing and synthesizing evidence on complex interventions

**DOI:** 10.1186/1745-6215-12-194

**Published:** 2011-08-16

**Authors:** Lori A Savard, David R Thompson, Alexander M Clark

**Affiliations:** 1 University of Alberta, 3rdFloor, Clinical Sciences Building, Edmonton, AB, T6G 2G3, Canada; 2 Department of Health Sciences and Department of Cardiovascular Sciences, University of Leicester, Leicester, LE1 6TP, UK

## Abstract

**Background:**

Despite favourable results from past meta-analyses, some recent large trials have not found Heart Failure (HF) disease management programs to be beneficial. To explore reasons for this, we evaluated evidence from existing meta-analyses.

**Methods:**

Systematic review incorporating meta-review was used. We selected meta-analyses of randomized controlled trials published after 1995 in English that examined the effects of HF disease management programs on key outcomes. Databases searched: MEDLINE, EMBASE, Cochrane Database of Systematic Reviews (CDSR), DARE, NHS EED, NHS HTA, Ageline, AMED, Scopus, Web of Science and CINAHL; cited references, experts and existing reviews were also searched.

**Results:**

15 meta-analyses were identified containing a mean of 18.5 randomized trials of HF interventions +/- 10.1 (range: 6 to 36). Overall quality of the meta-analyses was very mixed (Mean AMSTAR Score = 6.4 +/- 1.9; range 2-9). Reporting inadequacies were widespread around populations, intervention components, settings and characteristics, comparison, and comparator groups. Heterogeneity (statistical, clinical, and methodological) was not taken into account sufficiently when drawing conclusions from pooled analyses.

**Conclusions:**

Meta-analyses of heart failure disease management programs have promising findings but often fail to report key characteristics of populations, interventions, and comparisons. Existing reviews are of mixed quality and do not adequately take account of program complexity and heterogeneity.

## Background

Heart failure (HF) disease management programs are common in North America, Europe, and Australia [1,2]. These services provide care to optimize pharmacological regimen and support medication management and effective self-care. Programs have been widely introduced following recommendations from international clinical guidelines [1,3,4] but a number of recent and comparatively large trials have found no or small benefits from programs [5-10]. These inconsistencies have been explained by design issues rather than biases, reporting inadequacies or differences in actual effects [11,12]. However, recent results from the United States of the Medicare Health Support Pilot Program (MHSPP) [13] provide corroboration that program effects are poorly understood. This independent randomized trial of nine disease management programs with 30,000 patients with heart failure and diabetes concluded that programs did not decrease mortality, frequency of hospitalization, costs, or improve self-care, self-care efficacy, or mental and physical health [13].

These results raise questions about what clinicians should do in the light of contradictory evidence from trials and meta-analyses. When results from trials differ, it should not be concluded that an intervention is ineffective because most trials are underpowered to identify true effects [14]. Meta-analyses can overcome this lack of power but are as prone to reporting and design flaws as any other type of research design [15]. Though findings from meta-analyses frequently influence guidelines, like any other research design, as the recent PRISMA guidelines acknowledge, systematic reviews can vary widely in quality [16,17].

Thus, the methods and overall quality of meta-analysis are of great importance. Despite this, there has been no systematic appraisal of the quality of meta-analyses of heart failure management programs to date. This is particularly important given the increasing awareness of the complexity and diversity of these programs [18]. To evaluate the strength of evidence from current meta-analyses of these programs, we appraised the nature and quality of evidence from existing published meta-analyses of HF disease management programs.

## Methods

Meta-review was used to identify and appraise evidence from published meta-analyses of heart failure disease management programs or approaches. Meta-review appraises and synthesises findings from systematic reviews, in this instance, from meta-analyses [19]. The approach has evolved in response to the growing number of systematic reviews and the need to appraise quality of a review before application to practice and policy, for example via PRISMA [17].

Meta-review follows similar principles to systematic review [19]: it involves a comprehensive and detailed search of the literature for relevant studies with quality assessment to assess for bias, transparency, and comprehensiveness [19]. As with traditional systematic review, in meta-review, validation of quality by a second, independent reviewer is important to reduce potential for bias [19].

A comprehensive search was done to identify meta-analyses of randomized controlled trials published in English that examined the effects of HF disease management programs on key outcomes. To be included, reviews had to have a detailed and comprehensive search strategy (as identified by: naming of databases *and *years of searching *and *example or actual terms), contain data on study quality and make reference to synthesis of findings either by pooling data or rejecting the pooling of data. Due to changes in clinical practice, and to ensure some degree of congruence with contemporary clinical practice, we searched only for meta-analyses published after 1995, confined our search to reviews that contained comparisons of programs with usual care, and included samples of adults over the age of 18 years with confirmed diagnosis of HF. Meta-analyses of interventions that included patients with other forms of cardiac disease (such as cardiac rehabilitation or secondary prevention) that may have addressed heart failure disease management were not included due to the lack of data specific to heart failure populations in these reviews [20,21]. Finally, the meta-analyses had to contain extractable data for HF patients on mortality (all-cause or HF related), hospital (re)admission (all-cause and HF related)*, or *health-related quality of life.

For the purposes of the review, interventions were defined as HF management programs if they consisted of more than one recognized disease management component (medication optimization, lifestyle modification, or education) with the purpose of improving outcomes related to HF in patients with a confirmed diagnosis *or *were self-identified by the authors as constituting a program or analogous health service intervention beyond usual care for the treatment of HF.

A variety of electronic databases using a range of search terms (Table 1) were searched, including: MEDLINE, EMBASE, Cochrane Database of Systematic Reviews (CDSR), DARE, NHS EED, NHS HTA, Ageline, AMED, Scopus, Web of Science and CINAHL from 1^st ^January 1995 to July 31, 2008. In addition, reference lists and bibliographies of identified reviews were hand searched.

**Table 1:** Search terms used

Disease management-related	Heart failure-related
Disease management program (exp), manag(exp), educat(exp), Chronic disease (exp), program(exp), coach, usual care, counsel(exp), directive, organization, managed care programs, patient education, disease management (exp), care management (exp), randomized trial, program evaluation, evaluat(exp), meta-anal(exp), metaanal(exp), review(exp)	Heart failure(exp) chf

Exp: Exploded search

The primary screening was conducted independently by LS and AMC with abstracts/titles being screened fully. Full papers for potential inclusion were then screened by LS and AMC for detailed evaluation with disagreements regarding eligibility being handled with joint discussion between LS, AMC, and DRT.

Data were extracted onto a standardized data extraction template relating to: population, intervention, comparison, and outcome (PICO). This approach has been developed for optimizing evidence-based practice. Quality of each meta-analysis was assessed independently by LS and AMC using a standardized and valid measure of quality of systematic review (AMSTAR) [22].

## Results

**Figure 1:**
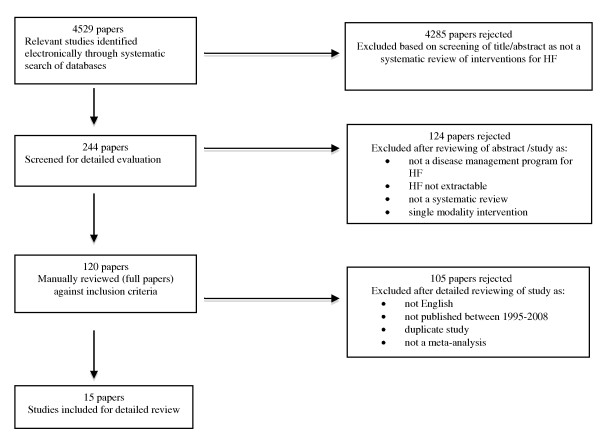
**Results of the systematic search strategy and study selection process**.

4529 potential articles were initially identified (Figure 1) but primary screening excluded 4285 papers. After reviewing the remaining papers (n = 244), 15 meta-analyses met the inclusion criteria (Table 2).

**Table 2:** Meta-analyses included in review

Review (Reference number)	Number of trials	N of total sample	Sex (% males)	Mean Age	SD Age	Age Range (Years)	AMSTAR Score
Koshman et al. [35]	12	2060	NR	NR	NR	58-80	7

Clark et al. [25]	14	4264	NR	NR	NR	57-75	8

Gohler et al. [32]	36	8341	37-99%	NR	NR	56-79	5

Jovicic et al. [24]	6	857	53-76%	NR	NR	56-76	7

Holland et al. [34]	30	NR	27-99%	NR	NR	56-80	4

Kim & Soeken [23]	4	NR	NR	NR	NR	NR to 64.0 to 81.6	6

Phillips et al. [31]	6	949	58%	73	NR	62-79	8

Roccaforte et al. [28]	33	3817	42%	73	NR	NR	8

Taylor et al. [27]	16	1627	23-86%	NR	NR	70-80	9

Whellan et al. [36]	19	5752	NR	NR	NR	56-80	2

Gonseth et al. [37]	54: 27 randomized and 27 non-randomized	3160	NR	Over 70 in most trials	NR	Not summarized	9

Gwadry-Sridhar et al. [33]	8	1239	37-58%	NR	NR	71 -80.3	6

McAlister et al. [30]	29	5039	NR	NR	NR	56-80	5

Phillips et al. [29]	18	3304	62%	NR	NR	NR	7

McAlister et al. [26]	11	2067	NR	NR	NR	63-80	5

The 15 meta-analyses (Table 2) contained a mean of 18.5 randomized trials +/- 10.1 (range: 6 to 36) and a mean of 3267.4 patients +/- 2184.0. Two reviews did not report sample size [9,23]. Overall quality of the meta-analyses based on AMSTAR criteria [22] was moderate but varied widely (Mean Score = 6.4 +/- 1.9; range 2-9). Main weaknesses in the reviews were lack of incorporation of study quality in conclusions and low detail regarding excluded studies (Additional file 1).

### Search Strategies

Most reviews searched for published and unpublished trials [9,24-31]; four identified that grey literature was searched [9,26,27,30]. Though only one review limited its search to English-only papers, [32] the overall quality of search strategies was moderate: three reviews described a full Boolean strategy [24,27,33] and eight provided a QUOROM-like flow chart [25,28-31,33-35]. Most reviews included an assessment of publication bias via a funnel plot [23-25,28,29,31,32,34].

### Populations

Mean age of the review population was calculated in two reviews [28,31] (both mean age: 73 years) with the oldest reported mean age being 81.6 [23]. Seven reviews [23,26,27,30,33,34,36] reported an upper age limit of 80 years. The lowest mean age reported was 56 by five reviews by way of inclusion of the same trial [24,30,32,34,36]. Two additional reviews reported lower mean age limits of 57 and 58 [25,35] but none presented data on standard deviation of ages.

Six reviews [23,25,26,30,35,36] provided no data on the sex of the participants in the trials. Co-morbidities and characteristics of study populations were frequently not reported with particular weaknesses in reporting of medication treatments (Table 3). Of the four studies that did report co-morbidities, [26,31,36,37] hypertension, diabetes, chronic obstructive lung disease, and coronary artery disease were most common.

**Table 3:** Select population characteristics

Reporting of population characteristics		
No information on co-morbidities	7/15	[24,25,29,30,32,33,35]

Incomplete or no data on NYHA Classification	10/15	[9,23,24,26,27,29,30,33,35-37]

No data on NYHA Classification	4/15	[23,26,27,30]

Range of NYHA Classification only	6/15	[24,25,28,31,32,37]

Information on LVEF	6/15	[25,29,31,34,36,37]

Summaries of ACE-I and BB medication treatments	3/15	[32,36,37]

^a^NYHA, New York Heart Association

^b^LVEF, Left Ventricular Ejection Fraction

^c^ACE-I, Ace-Inhibitor

^d^BB, Beta Blocker

### Interventions

#### Definitions of trials

Reviews most frequently used operationalised definitions (Table 4) to guide inclusion of interventions, though only three used definitions involving approach, personnel, setting, and content [23,26,27]. The foci of reviews differed markedly, for example, reviews specified interventions provided only in particular settings, [23,25-27] or without reference at all to content [25,34,37].

**Table 4:** Definitions of trials and characteristics of interventions actually included

Definitions of interventions included in reviews		
No definition	1/15	[33]

Operationalized definition	11/15	[23-27,30-32,34,36,37]

Definitions incorporate approach, personnel, setting and content	3/15	[23,26,27]

Concept of a disease management program	5/15	[26,28,30,31,36]

Concept of comprehensive treatment approach	6/15	[23,24,27,29,34,37]

Interventions provided in particular settings	4/15	[23,25-27]

**Intervention Settings**		

Five settings/modes of provision	2/15	[29,34]

Four settings	7/15	[27,30,31,33,35-37]

Three settings	4/15	[24,26,28,32]

Single or comparable setting	2/15	[23,25]

**Type of setting**		

All settings	2/15	[29,34]

Hospital (Pre-discharge)	10/15	[23,27-29,31,33-37]

Hospital and home-based components	9/15	[27-29,31,33-37]

Home and community	9/15	[27-29,31,33-37]

At home	13/15	[24,26-37]

Out-patient	13/15	[24,26-37]

Telephone	11/15	[24,25,27,29-35,37]

Remote provision	5/15	[25,29,30,34,36]

**Professionals**		

Nurse-led	11/15	[23,24,26,28-32,34,36,37]

'Multi-disciplinary teams'	10/15	[23,25,26,28,30,32-35,37]

Physician involvement via cardiologist or GP	9/15	[26,28-32,35-37]

Both cardiologist and GP	3/15	[26,30,36]

Pharmacist	6/15	[28-30,32,35,37]

#### Interventions included

Interventions included in reviews mostly spanned three to five patient settings or modes of provision; only two were focused on interventions using single or comparable settings or mode of provision [23,25]. Interventions included in reviews were wide ranging (Table 4) in terms of number and type of settings and locations. For example, nine reviews included programs with both hospital and home-based components [27-29,31,33-37] and two reviews included studies that employed interventions in all settings [29,34]. Nurses were the most frequent providers of care through 'multi-disciplinary team' interventions. Additional physician involvement via cardiologist or general practitioner was identified in nine reviews [26,28-32,35-37] though three reviews involved both general practitioners and cardiologists [26,30,36]. All reviews but one [24] identified other personnel involved, for example: pharmacist or pharmacist collaboration [28-30,32,35,37].

#### Program Content

The reviews specified a mean of 1.13 essential components of content (range 0 to 3). Interventions were described in terms of content using general descriptors, such as education, self-care, discharge plan, and medication support. Reviews most commonly stated that interventions had to have three or four component items though reviews could extend to five or more content components [26,30,37]. Educational and monitoring interventions were the most commonly identified elements. Other components included support at hospital discharge, medication review, and social support. Hence, a degree of overlap existed across settings. For example, a systematic review may focus on a nurse-led hospital-based intervention yet offers home visits, telephone support, and follow-up with a general practitioner [23].

Obtaining data on usual care was noted to be problematic [23,27-29,32,35] and the care provided to comparison groups was poorly defined (Table 5). For example, in seven of twelve trials in one review, descriptions of care were omitted entirely [35].

**Table 5:** Components of interventions included and trial quality

Number of components		
3-4	8/15	[23,24,27-29,31,32,35]

> 4	3/15	[26,30,37]

Educational components	13/15	[23,24,26-35,37]

Monitoring via home visits or phone	13/15	[23,24,26-32,34-37]

Support at hospital discharge	10/15	[23,24,26,28-32,36,37]

Medication review	9/15	[24,26-28,30-32,35,37]

Social support	5/15	[26,27,29,30,37]

**Comparison Groups**		

No information	11/15	[23-26,29-31,33,34,36,37]

### Outcomes

The follow-up period was 3 to 12 months in six reviews [24,27,29,31,33,36]. Three studies reported beginning follow-up periods at three months but the upper limit extended to 16, 18, and 22 months [25,32,28]. Other reviews did not report length of follow-up [34] or did not report follow-up periods [23].

### Within review pooling of outcomes

The meta-analyses pooled data on: all-cause mortality as primary and secondary outcomes. (Table 6) Other outcomes pooled included all-cause (re)admission, HF mortality, HF (re)admission, quality of life, and cost. Data were pooled using random [25,28,30,32-35] and fixed effect models of analysis [24,27] or both methods [26,29,31,37] if significant statistical heterogeneity was identified.

**Table 6:** Effect sizes of primary outcomes of reviews (95% Confidence Intervals)

Review (reference number)	All cause mortality	All cause re-hospitalization	HF-related hospitalization
Koshman et al. [35]	OR 0.84 (0.61-1.15)	OR 0.71 (0.54-0.94)	OR 0.69 (0.51-0.94)

Clark et al. [25]	RR 0.80 (0.69-0.92)	RR 0.95 (0.89-1.02)	RR 0.79 (0.69-0.89)

Gohler et al. [32]	RD 0.03 (0.01-0.05)	RD 0.08 (0.05-0.11)	NA

Jovicic et al. [24]	OR 0.93 (0.57-1.51)	OR 0.59 (0.44-0.80)	OR 0.44 (0.27-0.71)

Holland et al. [34]	RR 0.79 (0.69-0.92)	RR 0.84 (0.79-0.95)	RR 0.70 (0.61-0.81)

Kim & Soeken [23]	NA	OR 0.87 (0.69-1.04)	NA

Phillips et al. [31]	RR 0.80 (0.57-1.13)	RR 0.91 (0.72-1.16)	NA

Roccaforte et al. [28]	OR 0.80 (0.69-0.93)	OR 0.76 (0.69-0.94)	OR 0.58 (0.50-0.67)

Taylor et al. [27]	OR 0.86 (0.67-1.10)	NA	OR 0.52 (0.39-0.70)

Whellan et al. [36]	NA	NA	NA

Gonseth et al. [37]	RR 0.75 (0.59-0.96)	RR 0.88 (0.79-0.97)	RR 0.70 (0.62-0.79)

Gwadry-Sridhar et al. [33]	RR 0.98 (0.72-1.34)	RR 0.79 (0.68-0.91)	NA

McAlister et al. [30]	RR 0.83 (0.70-0.99)	RR 0.84 (0.75-0.93)	RR 0.73 (0.66-0.82)

Phillips et al. [29]	RR 0.87(0.73-1.03)	RR 0.75 (0.64-0.88)	RR 0.65 (0.54-0.79)

McAlister et al. [26]	RR 0.94 (0.75-1.19)	RR 0.87 (0.79-0.96)	NA

RR: Risk Ratio; RD: Risk Difference; OR: Odds Ratio

Out of 13 reviews, 6 identified statistically significant improvements in all cause mortality [25,28,30,32,34,37] though all 13 reviews identified trends favouring programs over control. Effect sizes varied from 3% to 25% but were mostly clustered around 15% to 20%. Larger benefits were more evident in terms of hospitalisations. All 9 reviews that measured changes in HF-related hospitalizations [24,25,27-30,34,35,37] identified significant reductions in admissions with reductions in risk ranging from 30% to 56%. Out of 13 reviews, 10 reviews [24,26,28-30,32-35,37] identified reductions in all-cause readmission with reductions in risk ranging from 8% to 41% with most clustered around 15% to 25% reductions in admission. Seven reviews extracted data on quality of life or health-related quality of life [27-31,33,35]. (Table 7) The majority did not pool outcomes due to high levels of heterogeneity [27,28,33,35] or lack of data [30]. However, two reviews identified insignificant trends favouring quality of life improvements after pooling [29,31].

**Table 7:** Direction of effects Quality of Life

Review	Measures	Pooling (Y/N)	Result of pooling
Phillips et al. (2005) [31]	NHP MLHF HFSBS	Y (5/6 studies reported QOL)	+ Intervention: 30.6 ± 20.7% VS. control: 19.3 ± 12.6%, p = 0.13

Gwadry-Sridhar et al. (2004) [33]	SF-36	N (4/8 studies reported HRQOL; precluded pooling)	RNPH

Koshman et al. (2008) [35]	MLHF COOP/WONCA SF-36 EQ-5D CHFQ	N (7/12 studies reported HRQOL; precluded pooling)	RNPH

Roccaforte et al. (2005) [28]	MLHF SF-36	N (16/33 studies reported QOL; varying presentation of results precluded pooling)	RNPH

Taylor et al. (2005) [27]	MLHF QLHFQ CHFQ Time trade off method (?)	N (8/16 studies reported HRQOL; varying results)	RNPH

Phillips et al. (2004) [29]	MLHF NHP HFSBS SF-36	Y (5/18 studies reported HRQOL)	+ Intervention: 25.7% [95% CI, 11.0%-40.4%] VS. control: 13.5% [95% CI, 5.1%-22.0%]

McAlister et al. (2001) [26]	NR	N (5/11 studies reported HRQOL)	Insufficient data

HRQOL = Health-related quality of life; QOL = Quality of life

MLHF = Minnesota Living with Heart Failure Questionnaire; COOP/WONCA = Dartmouth Primary Care Cooperative Research Network/World Organization of National Colleges, Academics and Academic Associations of General Practitioners/Family Physicians; SF-36 = 36-Item Short-Form Health Survey; EQ-5D = EuroQol-5 Dimensions form

NHP = Nottingham Health Profile; HFSBS = Heart Failure Self Care Behaviour Scale; QLHFQ = Quality of Life in Heart Failure Questionnaire; CHFQ = Chronic Heart Failure Questionnaire

RNPH: Results not pooled due to heterogeneity

+ = Non-significant trend favoring intervention

++ = Significant trend favoring intervention

Due to the limited reporting of interventions and control groups and the diversity of trials included in the reviews, it is not appropriate to pool outcomes from the meta-analyses here. This is important because findings from interventions that are excessively heterogeneous should not be pooled. Particularly, this was the case with these meta-analyses that varied and/or contained unclear data pertaining to a wide range of factors and strata of programs, for example, relating to clinical populations, providers, location, mode of delivery, numbers of components, and length. These multiple ambiguities made pooling, sensitivity analysis, and meta-regression inappropriate [38-40].

### Handling of uncertainty in the reporting of review results

Trial quality was inconsistently taken into account when formulating conclusions and was not addressed in most reviews. Statistical heterogeneity was discussed in most reviews though clinical and methodological heterogeneity was consistently neglected (Table 8). Sensitivity analyses were carried out around a diverse range of elements, including study quality, [23,28-30,34,35,37] size, [37] and publication status [34]. Intervention-type, [26,28] follow up, [26,30] diagnoses, [23] and elements of interventions related to: components, [23,26] complexity, [31] and provider-type [28,37]. Three reviews selected factors a priori for sensitivity analysis [26,30,37]. Sub-analyses were undertaken around 'general' program features, [32] setting, [26,30,34] home-visit or telephone contact, [26] and discharge planning [29,31].

**Table 8:** Trial quality and heterogeneity

Conclusions	
**Quality**	

Trial quality taken into account	[35,37]

Trial quality mentioned	[23,24,27,33]

**Heterogeneity**	

Discussed	[27,28,30,32,34,35,37]

Statistical Assessment	[23-35,37]

Cochran's Q test and I^2 ^statistic	[23-35,37]

Clinical acknowledged	[24,25,32]

Methodological acknowledged	[26,33,35]

## Discussion

This meta-review is the first of meta-analyses of HF disease management programs and conveys the challenges of performing meta-analyses of complex health services interventions. Overall, quality of the reviews was moderate though very mixed across reviews - this quality is important to consider when deciding whether review findings should guide practice and guidelines [22,41,42].

Based on the consistency and size of effect sizes identified by the meta-analyses, it would immediately appear reasonable to conclude either that, in generality, programs work or that programs of various types work [43]. However, this meta-review supports concerns that populations, programs, and analyses of these programs are inconsistently and poorly described [44,45]. For example, studies were poorly described in terms of populations and treatments with only one-fifth of reviews defining programs comprehensively in terms of approach, personnel, setting, and content. Even with the use of operationalised definitions to guide study selection in reviews, findings from interventions with very diverse characteristics and populations were pooled and, though mentioned in reviews, the implications of trial quality or statistical, clinical or methodological heterogeneity were seldom actually taken into account in analyses. No progress over time was evident in quality of reporting. Hence, reviews continue to focus on the results of study pooling over issues related to program complexity and heterogeneity.

Why might program complexity and heterogeneity be comparatively neglected in comparison to the findings of reviews? Firstly, this emphasis is understandable due to limitations in methodology. Complex interventions are often poorly described in published manuscripts [46] and it is well known that HF disease management programs are complex and diverse [43,45,47]. Current statistical and methodological techniques to describe and analyse such interventions in systematic review remain rudimentary [48]. Current meta-analyses also predate the existence of a taxonomy to classify HF disease management programs [18] and more extensive CONSORT reporting requirements for non-pharmacological trials [49].

Secondly, scientific findings that are more positive are more likely to be published in higher impact journals and cited more often in guidelines [50,51]. This reduces incentives to qualify results to take account of 'messy' issues related to program diversity and heterogeneity and fosters a disproportionate emphasis on positive findings without qualification [52] or recognition of how elements of context may moderate intervention effects [53]. This tendency may be combined with a wider perceived political need to champion multi-disciplinary health services interventions to attain greater recognition and usage of such interventions in healthcare systems seen to favour pharmacological interventions and biomedicine [54].

However, paradoxically, ignoring complexity and heterogeneity may actually reduce knowledge translation. This follows because uptake is likely to be reduced by unclear descriptions of what programs and comparison groups consist of, lack of clarity over likely benefits in important patient groups (for example: the effects of both age and sex on program outcomes are not known), and lack of specificity in findings regarding key program characteristics [16,53].

In future reviews, programs should be described comprehensively using systematic classification methods [18]. More sophisticated taxonomies are needed to fully capture the deeper characteristics of programs [48]. These should be used in future reviews to describe programs comprehensively and the effects of clinical, methodological, and statistical heterogeneity - as per PRISMA guidelines - must be formally taken into account in methods and conclusions [15]. Future trials should report key elements of populations, interventions, comparison group, and outcomes in accordance with the modified CONSORT statement for non-pharmacological trials [49]. These factors should be incorporated and reported comprehensively in meta-analyses. Findings from meta-analyses should be evaluated prior to application to practice and policy with review quality being assessed using valid quality criteria [15].

In terms of limitations, as with any review, this meta-review was constrained by the quality of reporting of the component studies. The data presented here are descriptive because it was inappropriate to synthesise outcomes to generate pooled effect sizes due to the wide diversity of programs subsumed in the reviews and the lack of comprehensive reporting in the reviews of intervention, comparator groups, and population characteristics [55,56]. As pivotal elements of programs, reporting of these components has to be clear and comprehensive if synthesis is to be undertaken.

## Conclusions

Meta-analyses of heart failure disease management programs have promising findings but often fail to report key characteristics of populations, interventions, and comparisons. Existing reviews are of mixed quality and do not adequately take account of program complexity and heterogeneity.

## Abbreviations

HF: heart failure

## Competing interests

The authors declare that they have no competing interests.

## Authors' contributions

AMC and DRT conceived the study, LAS and AMC extracted data from the reviews, and co-wrote the first and final drafts, DRT addressed issues of disagreement over interpretation of data and helped refine the manuscript. All authors read and approved the final manuscript.

## Supplementary Material

Additional file 1 **AMSTAR Quality Ranking of Included Studies**. Quality assessment ratings for each item on the AMSTAR tool for each review. Click here for file
