# Supplementary material for: A meta-review of evidence on heart failure disease management programs: the challenges of describing and synthesizing evidence on complex interventions
Source: Trials. 2011 Aug 16;12:194. doi: 10.1186/1745-6215-12-194 (PMC3174117; doi:10.1186/1745-6215-12-194)
Supplement: Additional file 1 — AMSTAR Quality Ranking of Included Studies. Quality assessment ratings for each item on the AMSTAR tool for each review. [file 1745-6215-12-194-S1.PDF]

**Study**	*A priori* design?	Independent data extractors?	Comprehensive literature search?	Status of publication as inclusion criteria?	List of included and excluded studies?	Characteristics of the included studies?	Documented quality of included studies?	Appropriate use of study quality in conclusions?	Appropriate methods?	Assessed publication bias?	Conflict of interests stated?	**AMSTAR TOTAL /11**
Koshman et al. [35]	Y	Y	N	N	N	Y	Y	Y	Y	N	Y	7
Clark et al. [25]	Y	Y	Y	Y	N	Y	N	N	Y	Y	Y	8
Gohler et al. [32]	CA	Y	N	Y	N	Y	N	N	Y	Y	N	5
Jovicic et al. [24]	CA	Y	Y	Y	N	Y	N	N	Y	Y	Y	7
Holland et al. [34]	CA	Y	Y	N	N	N	Y	N	Y	N	N	4
Kim & Soeken [23]	CA	Y	N	Y	N	Y	Y	N	Y	Y	N	6
Phillips et al. [31]	CA	Y	Y	Y	N	Y	Y	N	Y	Y	Y	8
Roccaforte et al. [28]	CA	Y	Y	Y	N	Y	Y	Y	Y	Y	N	8
Taylor et al. [27]	CA	Y	Y	Y	Y	Y	Y	Y	Y	N	Y	9
Whellan et al. [36]	CA	N	N	N	N	Y	N	N	Y	N	N	2
Gonseth et al.[37]	CA	Y	N	Y	Y	Y	Y	Y	Y	Y	Y	9
Gwadry-Sridhar et al. [33]	Y	Y	Y	N	N	Y	Y	N	Y	Y	N	7

McAlister et al. [30]	Y	Y	Y	Y	N	Y	N	N	Y	N	N	6
Phillips et al. [29]	CA	Y	N	Y	N	Y	Y	N	Y	Y	Y	7
McAlister et al. [26]	CA	Y	Y	Y	N	Y	N	N	Y	N	N	5

Supplementary Table 1 – AMSTAR Quality Ranking of Included Studies

Y = yes (1 point);
N = No (0 points);
CA = Can't answer (0 points);
N/A = Not applicable (0 points)
